# Accuracy of Ultrasound Scans as Compared to Fine Needle Aspiration Cytology in the Diagnosis of Thyroid Nodules

**DOI:** 10.7759/cureus.35108

**Published:** 2023-02-17

**Authors:** Mustafa Thaer Salman, Mustafa S AlGhazzawi, Eman A Al-Kamil, Sabrina Al-Salmi, Mustafa S Yousuf, Thair S Abdulla

**Affiliations:** 1 Anaesthesiology, Kettering General Hospital, Kettering, GBR; 2 Radiology, Al-Ahli Hospital, Doha, QAT; 3 Community Medicine, Hashemite University, Zarqa, JOR; 4 Anatomy, Hashemite University, Zarqa, JOR

**Keywords:** head &neck pathology, head and neck radiology, thyroid fnac vs ultrasound, thyroid cancer, : thyroid nodule, thyroid nodule. ultrasonography. thyroid cancer, benign and malignant thyroid nodule, ultrasound (u/s), thyroid fnac, fine needle aspiration cytology (fnac)

## Abstract

Introduction: Thyroid nodules (TNs) are among the more common findings on physical examinations. Due to the fear of the TN harboring malignancy and with the increasing incidence of thyroid cancer, ultrasound (US) scanning is used as an important diagnostic tool in the assessment of a TN. The American College of Radiology's Thyroid Imaging Reporting and Data System (TI-RADS) was established based on specific patterns composed of two or more features. According to the TI-RADS guidelines, a suspicious nodule by US findings should undergo fine-needle aspiration cytology (FNAC), in which results would guide further management.

Objective: This study was carried out to assess the accuracy of US as compared to FNAC in the diagnosis of a thyroid nodule.

Methodology: This retrospective study involved 213 cases that were sent for FNAC after having done a US scan of the thyroid. Data was gathered from all patient files that were referred for FNAC thyroid between 01/02/2018 and 30/06/2021 in Al-Ahli Hospital in the state of Qatar. The US scans were interpreted and reported according to the TI-RADS criteria. The FNAC samples were interpreted and reported according to the Bethesda System for Reporting Thyroid Cytopathology. Data were tabulated and analyzed with Excel (Microsoft, Redmond, WA, USA) and SPSS version 25 (IBM Corp., Armonk, NY, USA).

Results: The study showed that US had a sensitivity, specificity, positive predictive value (PPV) and negative predictive value (NPV) of 73.9%, 72.6%, 24.6% and 95.8%, respectively, with a significant association between the results of US and the results of FNAC (X^2^ (1, n = 213) = 20.295, p < .001) and a significant positive correlation (phi coefficient = .309, p < .001). In addition, the data showed that the odds for having a positive FNAC were 7.519 (95% CI: 2.811, 20.112) times greater for cases with positive US compared with cases with negative US. The relative risk of having a positive FNAC when the US was positive was 5.913 (95% CI: 2.440, 14.332) times greater compared to when the US was negative.

Conclusion: While our results showed that US cannot be solely relied on in diagnosing TNs, they did show that US can reliably rule out a malignancy in TNs. Recent studies have been showing increasing accuracy of US in diagnosing TNs and more studies are needed to explore this topic.

## Introduction

Thyroid nodules (TNs) are solid or fluid-filled lumps that form within the thyroid gland. The estimated prevalence by palpation is 3%-7% in some countries [1]. The prevalence is higher among randomly selected individuals by high-resolution ultrasonography where it may increase to 67% according to one study [2]. Thyroid nodules are always examined due to the fear of it being thyroid malignancy. The incidence of thyroid cancer is on the rise and is now the fifth most common cancer diagnosed in adult women worldwide and the second most common in women over 50 years of age [3,4]. The advancements in diagnostic technologies may be contributing to the increasing prevalence of TNs, but it may be explained by other traditional risk factors such as increasing age, insufficient iodine intake, exposure to radiation, and unhealthy lifestyles. This in turn increases the risk of obesity and metabolic syndrome, which are regarded as risk factors for TNs [5,6].

Several conditions can cause nodules to develop in the thyroid gland, including overgrowth of normal thyroid tissue, thyroid cysts, Hashimoto's disease, multinodular goiter and thyroid cancer [7]. Most TNs aren't serious and don't cause symptoms. However, a small percentage of TNs are caused by thyroid malignancy. This percentage is variable in different countries. A study in the United States found that only one out of every 20 clinically identifiable nodules turns out malignant [8]. Another study found that the proportion of thyroid cancer from TNs may reach up to 15% [9,10].

One of the important diagnostic tools in the assessment of TNs is ultrasound (US). It is currently the most accurate imaging modality for detecting TNs. It provides the best information about the shape and structure of nodules. Furthermore, it is useful as a guide in performing fine-needle aspiration (FNA) if required [11].

Different guidelines were proposed in order to help radiologists and clinicians readily recognize the sonographic patterns and classify nodules into categories. In 2009, the Thyroid Imaging Reporting and Data System (TI-RADS) was established based on specific patterns composed of two or more features. This model offers a standardized and simplified approach for radiologists to follow, with a good diagnostic performance of high sensitivity (88%), negative predictive value (88%) and accuracy (94%) [12]. However, radiologic findings alone are inconclusive. Therefore, according to the TI-RADS guidelines, a suspicious nodule by US findings should undergo FNA cytology (FNAC), in which results would guide further management [12,13].

In 2015, the American Thyroid Association (ATA) constructed new guidelines with a risk stratification model from very low suspicion to high suspicion for malignancy. It utilizes sonographic features based on the TI-RADS criteria. Patients with a TI-RADS score of 2 and 3 are considered low risk and are not routinely aspirated. This resulted in a reduction of the number of unnecessary FNAs [14]. A recent meta-analysis showed that the TI-RADS categories were a promising tool to differentiate between benign and malignant nodules, with a sensitivity and specificity of 0.79 (95% CI = 0.77-0.81) and 0.71 (95% CI = 0.70-0.72), respectively [15]. The objective of this study is to build up on this aspect of the literature, assessing the accuracy of thyroid US as compared to FNAC in the prediction of thyroid cancer.

## Materials and methods

This was a retrospective study of 213 cases that were sent for FNAC after a US scan of the thyroid. After gaining ethical approval from Al-Ahli Hospital, Doha, Qatar (EIC number EC2-2022), data was gathered from all patient files that were referred for FNAC thyroid between 01/02/2018 and 30/06/2021 at Al-Ahli Hospital. This amounted to 320 files. Of these, 25 cases had FNAC samples reported as inadequate and were therefore excluded from the study, leaving 295 cases. Of these, 82 did not have records of US scans of the thyroid and were therefore excluded from the study. This left us with a sample of 213 cases that had both a US scan and an FNAC thyroid.

The US scans were interpreted and reported by experienced radiologists according to the American College of Radiology's TI-RADS criteria. US features were scored as shown in Table 1 and, accordingly, the TI-RADS score was determined as shown in Table 2 [16].

**Table 1:** Thyroid Imaging Reporting And Data System (TI-RADS) criteria

Sonographic feature		Points
Composition	Cystic	0
	Spongiform	0
	Mixed cystic and solid	1
	Solid	2
Echogenicity	Anechoic	0
	Isoechoic or hyperechoic	1
	Hypoechoic	2
	Markedly hypoechoic	3
Shape	Wider-than-tall	0
	Taller-than-wide	3
Margins	Smooth	0
	Ill-defined	0
	Lobulated or irregular	2
	Extrathymoidal extension	3
Echogenic Foci	Non or large comet tail artefact	0
	Microcalcifications	1
	Peripheral (rim) calcifications	2
	Punctate echogenic foci	3

**Table 2:** Interpretation of Thyroid Imaging Reporting And Data System (TI-RADS) scoring

Score	Interpretation
0	TI-RADS 1 - benign
1 to 2	TI-RADS 2 - not suspicious
3	TI-RADS 3 - mildly suspicious
4 to 6	TI-RADS 4 - moderately suspicious
7 and above	TI-RADS 5 - highly suspicious

The FNAC samples were interpreted and reported by experienced histopathologists according to the Bethesda System for Reporting Thyroid Cytopathology. Histological results of the FNAC were classified as shown in Table 3 [17].

**Table 3:** The Bethesda System for Reporting Thyroid Cytopathology

Score	Interpretation
I	Unsatisfactory sample
II	Benign
III	Atypia or follicular lesion of undetermined significance
IV	Suspicion of follicular neoplasm
V	Suspicion of malignancy
VI	Malignant

For the purposes of this study, TI-RADS 1, 2 and 3 were considered negative US scan results as they are not very suspicious for malignancy, and are not a direct indication for FNAC. These cases are managed according to clinical suspicion, where clinical factors are taken into consideration rather than relying on the US result. On the other hand, TI-RADS 4 and 5 will be considered positive US scans as they hold high suspicion of malignancy and are a direct indication for FNAC, regardless of the clinical picture.

Bethesda I is an insufficient sample and as previously mentioned, these have been excluded from comparison in our study. Bethesda II is reported as benign. Bethesda III and IV are the "borderline" results and are considered for follow-up studies to further evaluate the nodule. However, Bethesda V and VI are considered malignant. Therefore, for the purposes of this study, Bethesda II, III and IV were considered negative and Bethesda V and VI were considered positive. 

Various demographic, clinical and sonographic criteria were considered in this research. The sensitivity, specificity, positive predictive value (PPV) and negative predictive value (NPV) were calculated for US scans of the thyroid. Odds ratio and relative risk were also calculated to compare US results to those of FNAC. The association between the various criteria considered in this study with the results of the US and FNAC was analyzed using chi-squared test. Data were tabulated and analyzed with Excel (Microsoft, Redmond, WA, USA) and SPSS version 25 (IBM Corp., Armonk, NY, USA).

## Results

The number of cases with the various criteria considered in the study are shown in Table 4. The age of the patients ranged between 17 and 70 (m = 42.62, SD = 10.84, n = 213). Of these, 174 (81.6%) were females. The number of cases with various features seen on US are shown in Table 5.

**Table 4:** Number of cases with various criteria considered in the study. TI-RADS: Thyroid Imaging Reporting and Data System; US: Ultrasound; FNAC: Fine Needle Aspiration Cytology.

Factor		n (%)
Gender (n = 213)	Male	39 (18.3%)
	Female	174 (81.7%)
Age Range (n = 213)	< 20	3 (1.4%)
	20 – 60	198 (93.0%)
	> 60	12 (5.6%)
Nodule firmness (n = 213)	Firm	2 (0.9%)
	Soft	211 (99.1%)
Regional lymphadenopathy (n = 213)	Present	9 (4.2%)
	Absent	204 (95.8%)
Rapidly growing (n = 213)	Yes	1 (0.5%)
	No	212 (99.5%)
Fixed or mobile (n = 213)	Mobile	213 (100%)
	Fixed	0 (0%)
History of neck radiology (n = 213)	Absent	213 (100%)
	Present	0 (0%)
TI-RADS Score (n = 213)	2	47 (22.1%)
	3	94 (44.1%)
	4	71 (33.3%)
	5	1 (0.5%)
US results (n = 213)	Positive	72 (33.8%)
	Negative	141 (66.2%)
Bethesda category (n = 213)	2	190 (89.2%)
	3	10 (4.7%)
	4	1 (0.5%)
	5	7 (3.3%)
	6	5 (2.3%)
FNAC results (n = 213)	Positive	12 (5.6%)
	Negative	201 (94.4%)
Vocal cord paralysis (n = 213)	Absent	213 (100%)
	Present	0 (0%)
Family history of thyroid cancer (n = 213)	Present	2 (0.9%)
	Absent	211 (99.1%)

**Table 5:** Number of cases with the different sonographic features seen.

Factor		n (%)
Echogenicity (n = 213)	Iso-echoic	114 (53.5%)
	Mildly hyper-echoic	22 (10.3%)
	Markedly hyper-echoic	3 (1.4%)
	Hypo-echoic	73 (34.3%)
	Markedly hypo-echoic	1 (0.5%)
Presence of cystic change (n = 213)	Cystic	49 (23.0%)
	Micro-cystic / Spongiform	16 (7.5%)
	Complex (solid + cystic)	71 (33.3%)
	Solid	77 (36.2%)
Halo Sign (n = 213)	Present	75 (35.2%)
	Absent	138 (64.8%)
Calcification (n = 213)	No calcification	146 (68.5%)
	Peripheral egg-shell calcification	22 (10.3%)
	Echogenic foci	34 (16.0%)
	Disrupted peripheral calcification	8 (3.8%)
	Micro-calcification	2 (0.9%)
	Globular Calcification	1 (0.5%)
Vascularity (n = 213)	Avascular	37 (17.4%)
	Peripheral vascularity	48 (22.5%)
	Mixed vascularity	106 (49.8%)
	Central vascularity	21 (9.9%)
	Intranodular vascularity	1 (0.5%)
Shape (n = 213)	Depth > Width	4 (1.9%)
	Other	209 (98.1)
Size range in CM (n = 213)	0.50-0.99	16 (7.5%)
	1.00-1.49	46 (21.6%)
	1.50-1.99	45 (21.1%)
	2.00-2.49	32 (15.0%)
	2.50-2.99	17 (8.0%)
	3.00-3.99	32 (15.0%)
	4.00-4.99	13 (6.1%)
	5.00-5.99	6 (2.8%)
	6.00 or greater	6 (2.8%)
Characteristic lymphadenopathy(n = 213)	Present	11 (5.2%)
	Absent	202 (94.8%)
Outline (n = 213)	Smooth / Well defined	183 (85.9%)
	Lobulated outline	11 (5.2%)
	Irregular outline	19 (8.9%)

There were 213 cases with both US and FNAC results. Of these, nine were positive for both tests and 138 were negative for both tests. Sixty-three cases had a positive US but negative FNAC. Only three cases had a negative US but positive FNAC. A significant association was found between the results of US and the results of FNAC (X2 (1, n = 213) = 9.6451, p = 0.0019) with a significant positive correlation (phi coefficient = 0.2128, p = 0.0019). The validity statistics for US as a diagnostic test for the diagnosis of thyroid carcinoma are shown in Table 6.

**Table 6:** Validity of ultrasound in the diagnosis of thyroid carcinoma.

Statistic	Value
Sensitivity	75.0%
Specificity	68.7%
Positive Predictive Value	12.7%
Negative Predictive Value	97.9%

In addition, the data showed that the odds for having a positive FNAC were 6.57 (95% CI: 1.7203, 25.1021) times greater for cases with positive US compared with cases with negative US. The relative risk of having a positive FNAC when the US was positive was 5.913 (95% CI: 1.6409 to 21.0344) times greater compared to when the US was negative.

## Discussion

As previously discussed, the current global consensus is that thyroid US on its own is insufficient to diagnose thyroid cancer. However, with the constantly evolving and advancing field of radiology, new studies are emerging that attempt to challenge this idea. A study conducted in Turkey in 2021 compared the reliability of the ATA and TI-RADS guidelines for thyroid US with FNAC results. It concluded that both guidelines can accurately predict malignancy, and may in fact eventually lead to a decrease in unnecessary FNAs [18]. Another study comparing the ATA, British Thyroid Association and TI-RADS showed that all three guidelines had sensitivities and NPV of over 90%, with ATA being the best at 98% and 95%, respectively [19]. In our study, we demonstrated a sensitivity, specificity, NPV and PPV of 75.0%, 68.7%, 97.9% and 12.7%, respectively.

Current literature shows large discrepancies between different studies. Shweel et al. conducted a similar study which showed sensitivity, specificity, NPV and PPV of 76.2%, 83%, 88.8% and 66.4%, respectively [20]. Another study by Trimboli et al. similarly showed a sensitivity of 61%, specificity of 83% and NPV of 83% [21]. Xing et al. and Wang et al. both demonstrated high sensitivities of 95.7% and 92%, respectively [22,23]. Similarly to our study, the former also demonstrated a very high NPV of 99.7%, as compared to the latter’s 63.1%. Zhang et al. demonstrated a sensitivity, specificity and NPV of 69%, 85% and 89%, respectively [24]. All of the aforementioned studies showed positive predictive values between 60% and 66.4%, with the exception of Wang et al. which showed a high PPV of 95%. Specificities ranged between 61% and 85%. The values our study has demonstrated appear to be average compared the current literature except for our very low PPV of 12.7%.

Russ et al. conducted a study comparing the efficacy of using US alone as opposed to US with elastography [25]. With the use of US alone, the study demonstrated a sensitivity, specificity, NPV and PPV of 70%, 92.4%, 87.6% and 80%, respectively. However, when combining both US and elastosonography together, the sensitivity and NPV went up to 98.5% and 99.8%, respectively. Subsequently, the study concluded that FNACs can be reduced by up to 34% using this combined approach. Shweet et al. and Trimboli et al. both demonstrated similar findings as well. The former demonstrated that the combined approach resulted in better performance, with sensitivity, specificity, NPV and PPV of 95.4%, 94.8%, 98.8% and 82.3% [20]. The latter showed the combined approach resulted in at least 97% sensitivity and 97% NPV [21]. The findings of these studies, among others, show promise in the possibility of eliminating or reducing the need of using FNACs for thyroid nodules.

The current gold standard for diagnosing thyroid cancer is FNAC. A meta-analysis of the Bethesda reporting system found that the sensitivity, specificity, NPV and PPV were 97%, 50.7%, 96.3% and 55.9%, respectively [26]. Another study comparing the effectiveness of TI-RADS criteria in US to the Bethesda reporting system of FNAC demonstrated that the Bethesda reporting system had a sensitivity, specificity, and accuracy of 90%, 94.3% and 91.1%, respectively [27]. These values are not significantly superior to the values demonstrated by ultrasonography alone in some of our previously mentioned studies. Furthermore, a study conducted in one center demonstrated a false negative rate of FNAC of 15%, concluding that the Bethesda risk stratification system often underestimates malignancy rates [28]. 

The results of our study should be considered in the context of the following limitations, one being our small sample size. A larger study would produce more reliable data, especially if conducted in a specialist center. A second limitation is the nature of the study itself. Patients are only referred for FNAC if they have suspicious findings on US. Therefore, it is difficult to assess the proportion of false negative cases, who may eventually have positive FNACs despite having negative US scans. Once again, a larger sample size may give more reliable data in light of this issue. Another limitation is the nature of healthcare in the region where this study was conducted, where lots of patients seek private healthcare, often outside of the country. This resulted in a large proportion of missing patient data in the hospital system. This is one of the reasons why so many patients had to be excluded from our study, as previously mentioned.

## Conclusions

US was shown to be a reliable tool in the assessment of TNs. There is a considerable amount of discrepancy between different literatures regarding this topic. Some studies and the current consensus suggest that US cannot be used without FNAC to diagnose TNs. Other studies, however, suggest that the advancement in US quality and techniques have led to US being up to par with FNAC in terms of accuracy. With the constant development and evolution of imaging techniques, we expect US scans to become more and more reliable. Highly specialized radiology centers with modern equipment and adequate experience may soon be able to, without the use of FNACs, achieve results that are on par with our current accepted standard. Therefore, we recommend that large studies be conducted in such centers to assess and compare the reliability of modern US scans in diagnosing TNs to the current gold standard of FNAC.
